# Nickel–titanium arched shape-memory alloy connector combined with bone grafting in the treatment of scaphoid nonunion

**DOI:** 10.1186/s40001-019-0380-y

**Published:** 2019-07-27

**Authors:** Pan-yu Zhou, Li-qiang Jiang, De-meng Xia, Jiang-hong Wu, Yuan Ye, Shuo-gui Xu

**Affiliations:** 1 Department of Emergency, Changhai Hospital, Second Military Medical University, Shanghai, China; 2 Department of Orthopedics, Changhai Hospital, Second Military Medical University, Shanghai, China

**Keywords:** Ni–Ti arched shape-memory alloy connector, Ni–Ti ASC, Bone grafting, Scaphoid nonunion

## Abstract

**Purpose:**

To summarize the techniques and clinical effectiveness in treating scaphoid nonunion with nickel–titanium (Ni–Ti) arched shape-memory alloy connector in combination with autologous iliac bone grafts.

**Methods:**

This study retrospectively analyzed 18 scaphoid nonunion cases treated with arched connectors with autologous iliac bone grafts. Based on scaphoid nonunion, 2 cases were classified as type II (fibrous union), 4 cases as type III (mild sclerotic union), 6 cases as type IV (moderate resorption and sclerosis), 5 cases as type V (severe bone resorption and sclerosis), and 1 case as type VI (pseudarthrosis formation). At the first 4, 8 and 12 weeks after the surgery, wrist anteroposterior, lateral X-ray were obtained, respectively, to evaluate bone healing. Patients who had not yet reached the standard of healing at 12 weeks after surgery would continue to receive additional appointments for follow-up visits, such as 14 weeks, 16 weeks, 18 weeks after surgery, until their imaging studies had achieved satisfactory bone healing. Clinical effectiveness was evaluated comprehensively, based on bone union time, Mayo wrist score, and visual analog pain score.

**Results:**

All 18 patients achieved satisfactory reduction and fixation with a mean union time of 4.2 months. Preoperative Mayo wrist score averaged 57.4 and average final postoperative follow-up was 91.4. On the other hand, mean preoperative VAS score was 6.8, and final postoperative follow-up average was 1.6. Mayo wrist score of the overall treatment effectiveness was excellent (90–100) in 12 cases, good (80–90) in 5 cases, and acceptable (60–80) in 1 case with zero poor (below 60) cases observed. Statistical analysis suggested that a statistically significant improvement in fracture healing, wrist function recovery and visual analog pain after surgery when compared to the scores of the patients before surgery.

**Conclusion:**

Using Ni–Ti arched shape-memory alloy connector in combination with autologous bone grafting provided a new modality to treat scaphoid nonunions in a less traumatic, convenient to operate and satisfactory manner in treatment outcomes, and thus is worthy of further application.

## Background

Scaphoid acts as an important hinge and lever that connects the distal and proximal carpal bones. It is the biggest among the carpal bones in the proximal row. Incidence of scaphoid fractures accounts to about 2% of body fractures and 60–70% of wrist fractures, the second highest following fractures of the distal radius [1]. Scaphoid is connected to the surrounding bones, the radius, lunate, capitate, trapezium and trapezoid, and 80% of its surface is covered with hyaline cartilage [2]. Due to the lack of periosteum coating, union of scaphoid fractures mainly depends on stage I healing, where a small amount of callus is formed. However, the biomechanical strength of the scaphoid in this stage is weak [3]. In addition, scaphoid receives majority of its blood supply via dorsal branches of the radial artery. These branches enter the scaphoid in a retrograde fashion along its dorsal ridge. As a result of the vulnerable blood supply, a high incidence of nonunion occurs after a scaphoid fracture [2, 4, 5]. Once scaphoid nonunion occurs, unstable deformities eventually cause carpal collapse of scaphoid nonunion arthritis in the dorsal insertion region of the wrist, resulting in weakness and pain during wrist movements and joint stiffness, thus severely affecting the quality of life of patients [4, 6–8]. These fractures were initially treated with single compression screws followed by improvements in treatment concept and internal fixation [9–11]. However, due to the limited stability and protracted cast immobilization, early activities of patients are often delayed, resulting in dissatisfactory clinical outcomes [5].

## Methods

### General information

Between June 2010 and December 2015, 18 patients with scaphoid nonunion were treated with Ni–Ti arched shape-memory connector (ASC; Seemine, Lanzhou Seemine SMA Co., Ltd., Gansu province, China) in combination with autologous iliac bone grafting we developed in-house. And the average number of bone nonunion patients treated in our clinical center was about 104 per year, including 3–4 cases of scaphoid bone nonunion each year. This study was approved by the institutional review board at our institution (No. 112016054, Second Military Medical University, Shanghai, China). Besides, making full preoperative communication with patients (or his/her legal guardian), enhanced mutual understanding and trust, and preoperative informed consent signature were obtained. Clinical complaints included local intractable wrist pain and discomfort with weakness during movements. Clinical examination found restrictions in wrist movements, and was most obvious in dorsiflexion, and point tenderness in the anatomical snuff box, radial styloid process—scaphoid joint or in the distal pole of the scaphoid on the volar side.

### Treatment methods

**Fig. 1:**
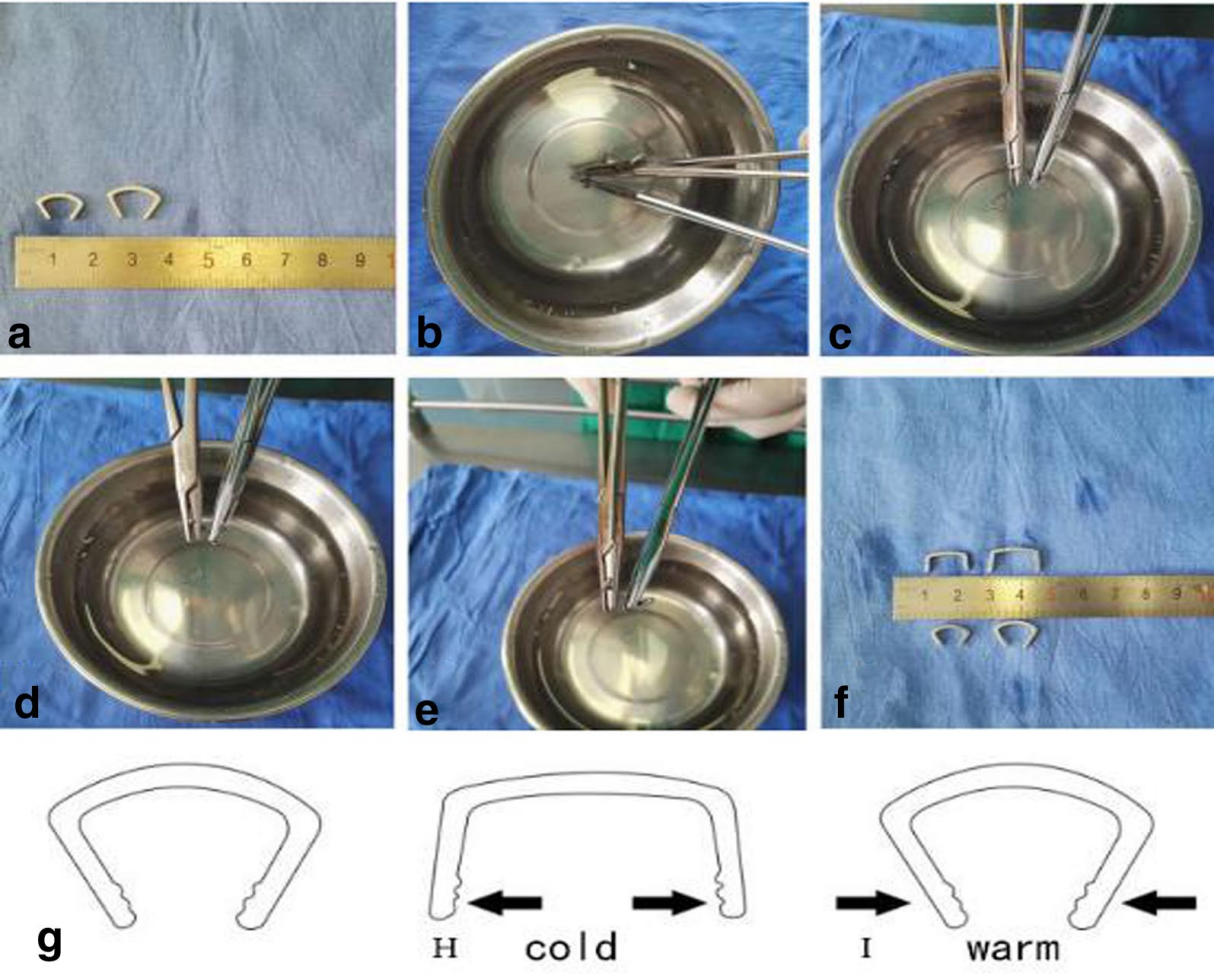
**a**–**f** Appropriate ASC model was selected based on the size of scaphoid and immersed in sterile salt water at 0–5 °C for 3–5 min and then flattened. **g**, **h** ASC is been flattened. **i** When meet with warm sterile salt water, ASC could offer sustain contract force

**Fig. 2:**
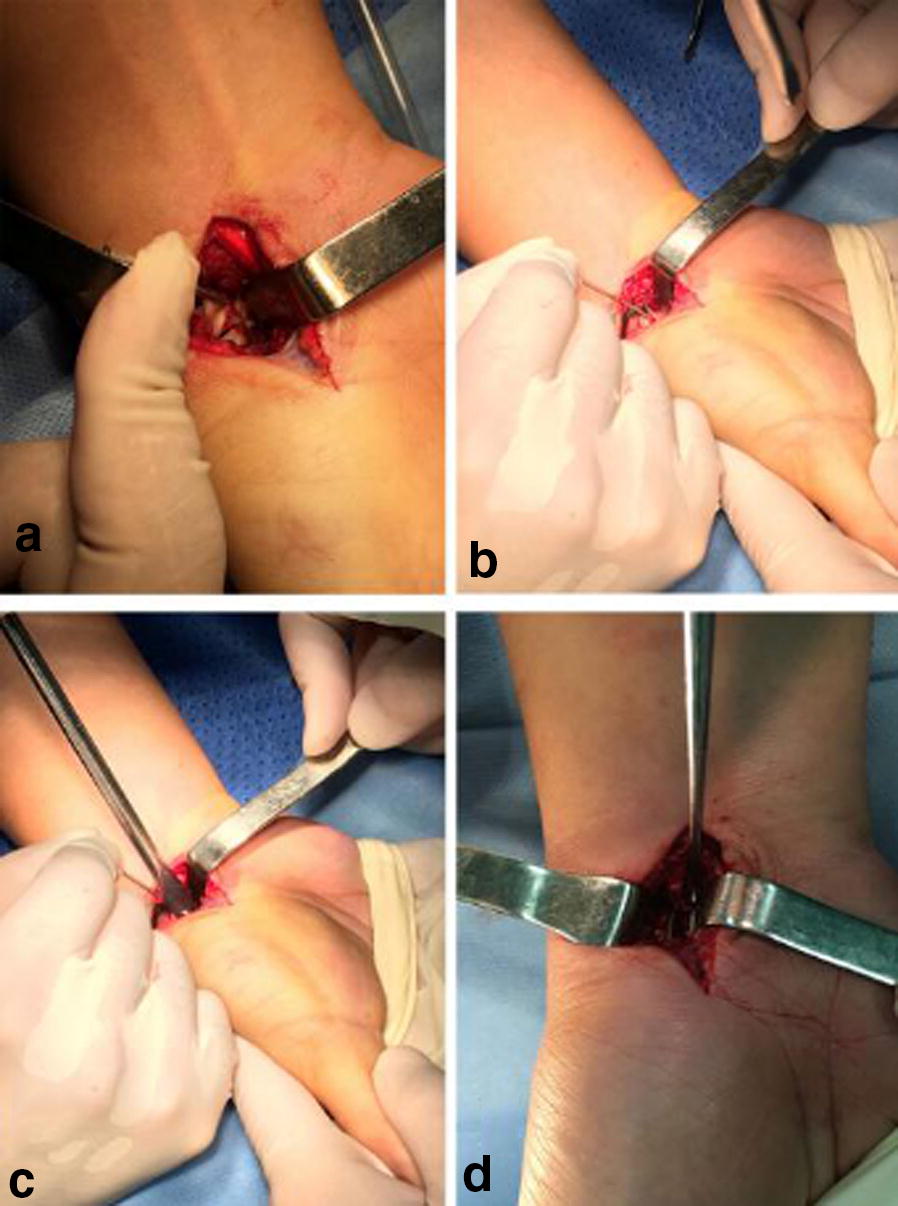
**a** Fibrous scar tissues and sclerotic calluses were removed until bleeding spots were seen from the fractured margins. **b** Graft of cancellous bone block with attached cortex was trimmed to match the size and shape of the scaphoid deficits and embedded in the deficit site. **c** Fracture margins were adequately filled up, fracture reduction was conducted, and then the preselected ASC was inserted. **d** Warm (35–40 °C) salt water was sprayed to gradually tighten the extended arms to produce compressive stress onto the nonunion margins, which became a valuable applied pressure to accelerate healing

**Fig. 3:**
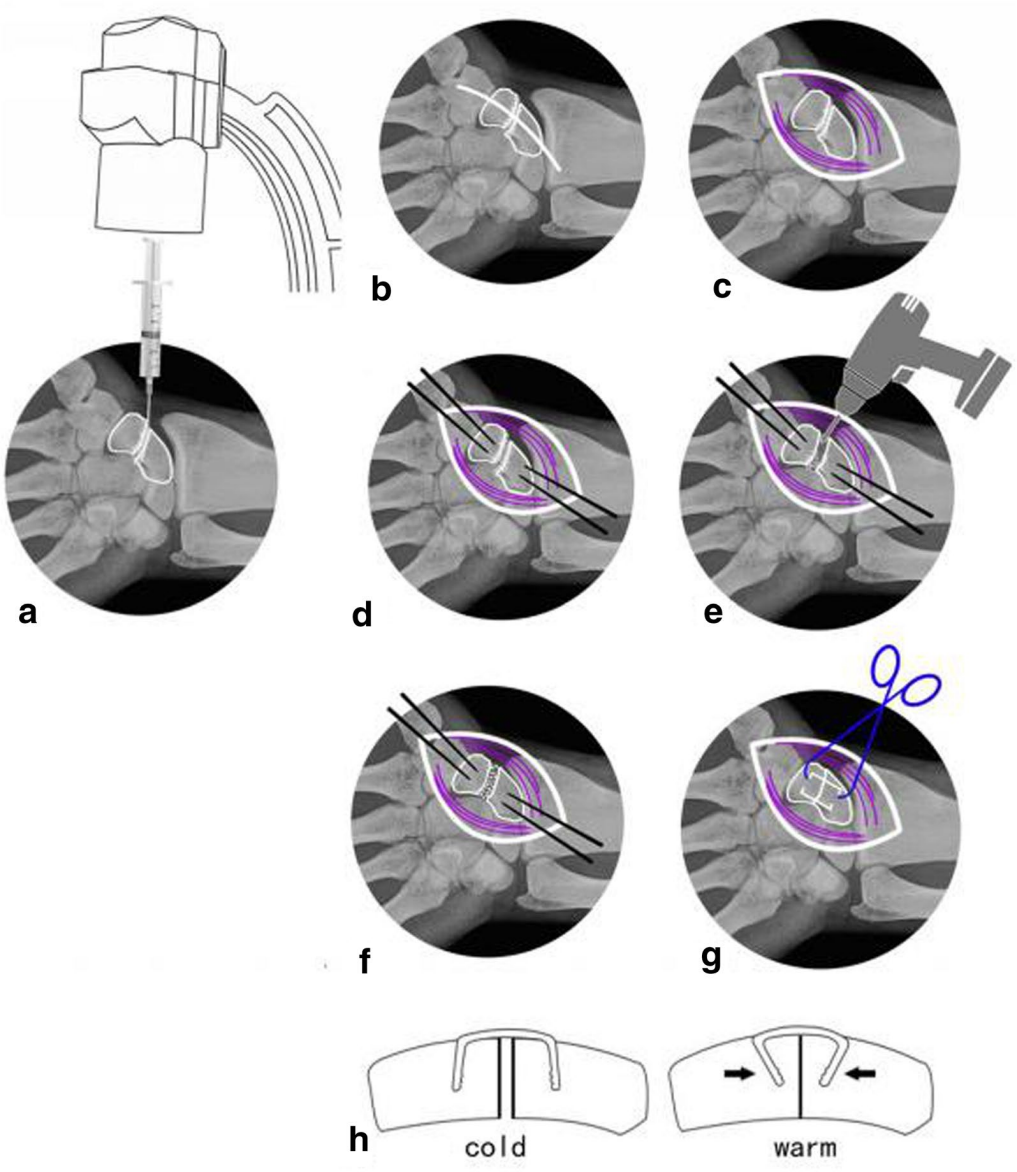
**a**–**c** Determine the location with a 5-ml syringe needle puncture under C-arm fluoroscopy and expose the scaphoid nonunion. **d** Proper locations for implantation were determined at proximal and distal poles of the bone and then, two φ1.2-mm K-wires were drilled in for poking. **e** Fibrous scar tissues and sclerotic calluses were removed through curetting using a scoop or drilling using an electric drill until bleeding spots were seen from the fractured margins. **f** The cancellous bone block graft with attached cortex was trimmed to match the size and shape of the deficits in the scaphoid; cancellous bone was used to adequately fill the treated proximal and distal fracture fragments followed by fracture reduction. Then, the graft of cancellous bone with cortex was embedded in the deficit site. **g**, **h** Choose the appropriate ASC and insert it, and then warm (35–40 °C) salt water was sprayed to gradually tighten the extended arms until their original shape was almost achieved

**Fig. 4:**
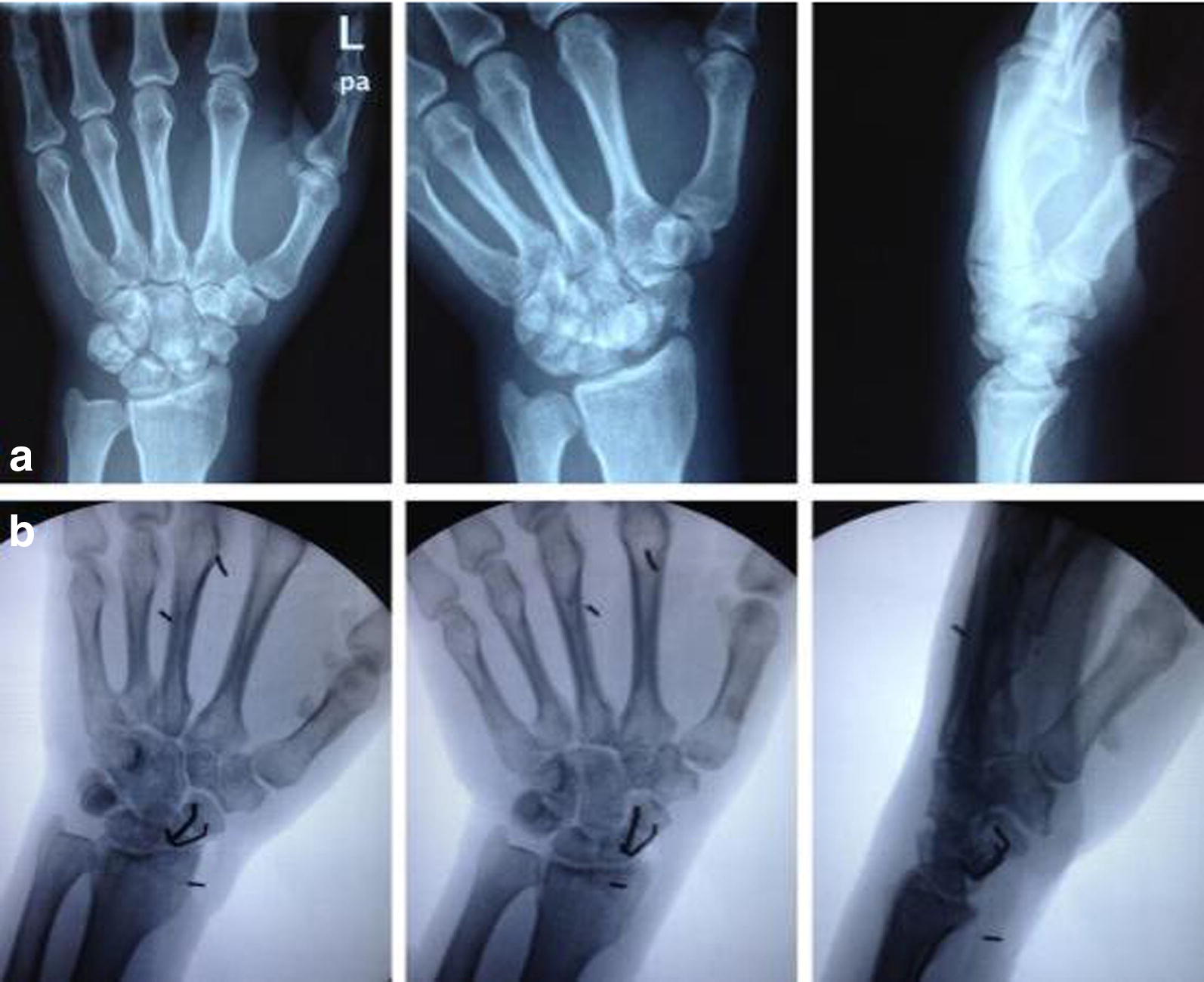
A 32-year-old male patient presented with left wrist pain and discomfort caused by a fall 6 months before followed by cast immobilization. **a** Preoperative X-ray on anteroposterior, butterfly and lateral views revealed old scaphoid fracture, bone resorption and pseudarthrosis formation at the fracture site. **b** Postoperative anteroposterior, butterfly and lateral X-ray demonstrated scaphoid fracture reduction, and ASC fixation was in place, and alignment was satisfactory

Under brachial plexus block anesthesia, ASC was applied to all internal fixations with autologous iliac bone grafts. Briefly, with patient supine, approximate location of the scaphoid nonunion was determined with a 5-ml syringe needle puncture under C-arm fluoroscopy, followed by a longitudinal incision of about 5 cm at the center of the nonunion site, and the scaphoid and the capitate were carefully exposed to facilitate subsequent fracture reduction. Precautions were taken to avoid separating the radial dorsal part of distal pole of the scaphoid during exposure, so as to prevent any vascular damages at the dorsal ridge. A small round blade (size 15) and a nerve stripper were used to identify the location of nonunion and the borders of scaphoid, and the appropriate ASC model was selected based on the scaphoid size. The selected ASC was immersed in sterile salt water at 0–5 °C for 3–5 min and then flattened as illustrated in Fig. 1. Proper locations for implantation were determined at proximal and distal poles of the bone and then, two φ1.2-mm K-wires were drilled in for poking while the selected ASC was immersed in iced salt water and kept ready for use. The location of the nonunion was exposed with the two inserted K-wires poking the fractured bone blocks. Fibrous scar tissues and sclerotic calluses were removed through curetting using a scoop or drilling using an electric drill until bleeding spots were seen from the fractured margins. Then, the tourniquet was loosened when necessary to evaluate blood supply to the fractured bone blocks. Main points of treatment to fractured margins included the following: (1) for newly formed nonunions with no obvious sclerosis at the fracture ends, an electric surgical knife (or small nucleus pulposus forceps) was used to burn-off (or bite-off) the soft tissues (or partially sclerotic bone) at the nonunion sites. (2) In relatively old nonunions, the fracture ends often become heavily sclerotic. Therefore, in addition to the methods above, drill grinding of the hardening bone is essential, but the cartilage shell should be carefully maintained to shape the bone graft into a wide-fracture surface and narrow-based defect filling. The cancellous bone block graft with attached cortex was trimmed to match the size and shape of the deficits in the scaphoid; cancellous bone was used to adequately fill the treated proximal and distal fracture fragments followed by fracture reduction. Then, the graft of cancellous bone with cortex was embedded in the deficit site. With towel forceps holding the distal and proximal poles to temporarily maintain reduction, K-wires were removed, the preselected ASC inserted, and then warm (35–40 °C) salt water was sprayed to gradually tighten the extended arms until their original shape was almost achieved. The compressive stress produced during the process was naturally transmitted to the nonunion margins and became a valuable applied pressure to accelerate healing. If necessary, another ASC may be implanted at an appropriate location to strengthen the fixation using the same method (as illustrated in Figs. 2 and 3). A satisfactory fracture reduction was identified by observations through C-arm fluoroscopy; these observations included smooth joint surfaces and the ASC in place (as illustrated in Fig. 4) with incision flushed, instruments and gauzes counted, volar carpal ligaments and joint capsule repaired. Then, the wound was sutured layer by layer and covered with dressing.

### Postoperative management and clinical effectiveness evaluation

A short arm thumb spica cast immobilization was applied for 4 weeks after the surgery. The plaster was cut over the wound to make a window to allow for convenient changing of dressing, once every 2 days. The sutures were taken out 2 weeks after the surgery, the cast was removed 4 weeks after the surgery, and rehabilitation was provided gradually to restore wrist function. At the first 4, 8 and 12 weeks after the surgery, wrist anteroposterior, lateral X-ray and unenhanced CT scan were obtained, respectively, to evaluate bone healing.

Indicators of clinical effectiveness included the following: (1) bone union time (fracture line blurring demonstrated on X-ray and local bone bridge formation identified on CT scan); (2) Mayo wrist function score (comparisons between preoperative and final follow-ups); (3) visual analog pain score (VAS score comparisons between preoperative and last follow-ups).

### Statistical analysis

All statistical analyses were conducted with the SPSS 19.0 software in which *x* ± *s* was used for quantitative data, paired *t* test was applied to test statistical significance between groups and *P* < 0.05 was considered as statistically significant.

## Results

This study included 14 males and 4 females aged 19–51 years who had injuries in the wrist; 7 cases had injuries on the left side and 11 cases on the right side, and excluded patients with mental disorders, or who had a clear cardiopulmonary impairment that could not tolerate surgery. All patients were advised to quit smoking, while seven of the patients had a smoking index of more than 100 and three of them had a smoking index of more than 400. One patient had type 2 diabetes for 7 years and was treated with oral hypoglycemic agents. All patients had no infection at the nonunion site, no malnutrition, no use of hormones, anticoagulants, or nonsteroidal anti-inflammatory drugs (NSAID). All injuries were caused by falls on the outstretched wrist. Based on the classification of scaphoid nonunion [12], 2 cases were classified as type II (fibrous union), 4 cases as type III (mild sclerotic union), 6 cases as type IV (moderate resorption and sclerosis), 5 cases as type V (severe bone resorption and sclerosis), and 1 case as type VI (pseudarthrosis formation). X-rays showed waist fractures in 13 cases and proximal fractures in 5 cases with varying degrees of bone resorption and sclerotic changes at fracture margins, of which some demonstrated a “humpback” deformity and obvious pseudarthrosis. A time frame between the occurrences of the fractures and surgeries ranged from 8 to 13 months, and all 18 patients achieved excellent reduction and fixation. Regular X-ray review showed that internal fixation was in place without loosening and displacement and CT scan showed that all patients achieved excellent bone union with the average healing time being 4.2 months (range 12–36 weeks, the average was 16.8 weeks, approximately equal 4.2 months). CT is most useful in evaluating an established scaphoid nonunion or malunion. Since plain radiographs are often unreliable, CT is preferred for confirming union after a scaphoid fracture [13, 14]. Average preoperative Mayo wrist score was 57.4 (range 51–72), while the average postoperative final follow-up score was 91.4 (range 78–100). Consistently, the average preoperative VAS score was 6.8 (range 5.5–9.6) and the average postoperative final follow-up score was 1.6 (range 0–3.5).

**Fig. 5:**
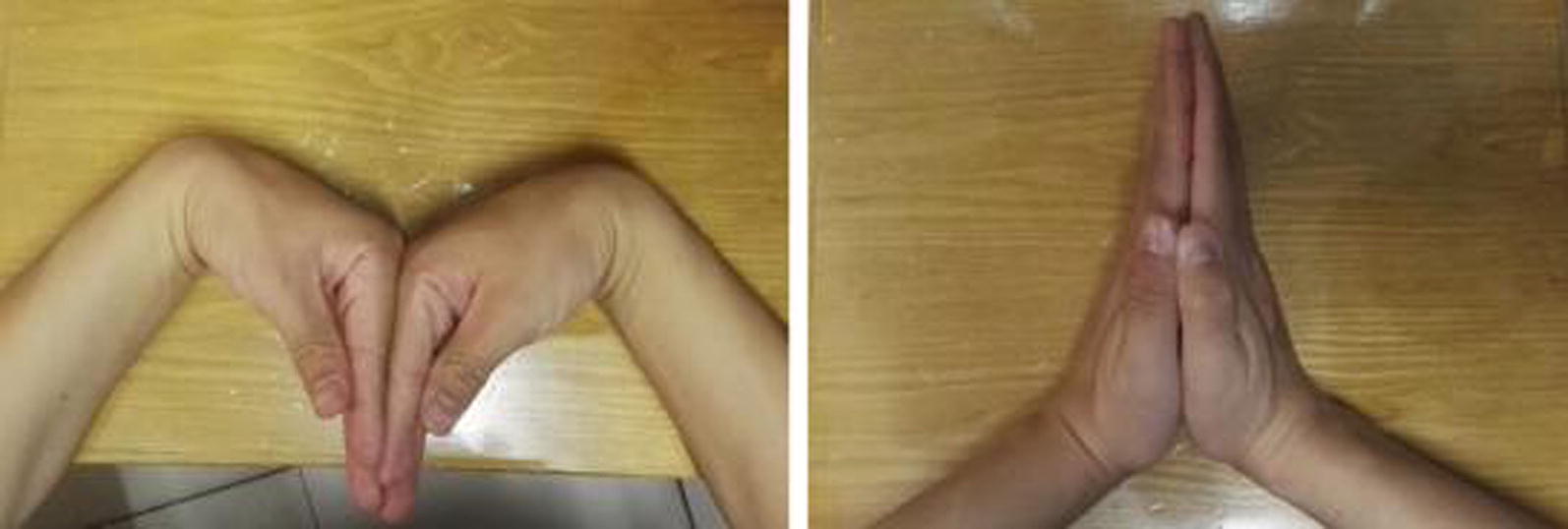
Good outcome in wrist function recovery

Based on the Mayo wrist score, the overall clinical effectiveness was also evaluated as excellent (90–100) in 12 cases, good (80–90) in 5 cases, and satisfactory (60–80) 1 case. Incisions of all the patients achieved stage I healing without complications. Intractable wrist pain, limited range of motion and other symptoms before surgery were significantly relieved in majority (*n* = 16) of the patients. Wrist function after surgery is illustrated in Fig. 5. Only 1 patient complained restriction in wrist movements in daily activities particularly while doing heavy lifting. CT scan, however, confirmed that bone union was achieved in this patient. Statistical analysis suggested a statistically significant improvement in fracture healing, wrist function recovery and visual analog pain after surgery when compared to the scores of the patients before surgery.

## Discussion

The scaphoid acts as a bridge connecting the forearm and the carpal bones, and is essential for maintaining normal wrist activities. The high incidence rate of scaphoid nonunion is closely associated with the unique anatomical structure of itself. Scaphoid has a relatively complex 3-dimensional morphology with its appearance described as “twisted peanut” [2, 15], 80% of its surface is coated with articular cartilage. When a guide pin of a cannulated needle or screw is inserted, it inevitably disturbs the joint structure disrupting the blood supply. Furthermore, the scaphoid receives majority of its blood supply through 2 main branches of radial artery [2, 15]. When fractures occur in the scaphoid waist or proximal pole, the intraosseous recurrent branch is frequently damaged, resulting in a high incidence of nonunion, delayed union or ischemic necrosis in the proximal pole. On the other hand, when internal fixation is performed to scaphoid fractures, the size and shape of the scaphoid, location of the fracture line and features of blood supply must be considered comprehensively and the operation conducted meticulously.

Additionally, mechanical instability is also one of the major risk factors for the occurrence of scaphoid fracture nonunion [1, 3]. High risk of fracture nonunion in the proximal pole or waist of the scaphoid is attributed to its long arm of force of the distal pole, a small adjunction area of the proximal pole and other biomechanical characteristics. In addition, the use of inappropriate methods to fix fractures, fragment displacement or slight movement may further prevent reconstruction of vascular ingrowth, resulting in acceleration of bone resorption and local sclerosis formation, eventually leading to nonunion.

Therefore, surgical treatment of scaphoid fractures must focus on solving 3 critical issues. *First* is blood supply reconstruction at the fracture site; *second* is debridement (i.e., to remove all necrotic tissues) and replacement with bone conductive and inductive matrix; and *finally* stabilize and reset fracture fragments. There are several fatal flaws with the use of cannulated screws to fix scaphoid nonunions: (1) mechanical properties of the fixation are relatively weak. The screw has a tendency to loosen or break, even risking another fracture while [16]. (2) When treating scaphoid nonunion, a percutaneous cannulated screw is only applicable for I–III-degree scaphoid nonunion with mild symptoms, provided that blood supply in the distal pole of the fragment is adequate [17]. (3) The two ends of the fractured bone of scaphoid nonunion have to be large enough, and after local sclerosis scraped, an adequate mass of the remaining bone is also required to ensure that the screw threads do not cross the grafting area, so as to prevent failure in compressive fracture fixation [18].

In contrast, a number of advantages can be noted in using Ni–Ti ASC to treat scaphoid nonunions [18–20]. (1) During the surgery, there is flexibility in choosing the matching specification of the ASC based on the size of fracture fragments and the location of fracture line. Also, the ASC can be pre-bent and shaped in line with the expected position for implantation, and it is easy to operate with a smooth learning curve and achieve a high success rate. (2) With a small incision on volar approach, only the fracture site is exposed, where reduction is performed under direct vision and the operating area of internal fixation insertion is relatively small. The blood supply in the fracture site is sufficiently protected, which benefits fracture healing. (3) More importantly, as the arched connector is made of nickel–titanium shape-memory alloy material, after implantation it constantly generates memory effect, which provides valuable applied pressure at the fracture site, helps stabilize the nonunion margins and accelerates healing. While several attempts and repeated X-ray fluoroscopy are often required for cannulated screws internal fixation, it is hard to confirm the optimal position and length for screw placement compared with ASCs implantation, which should fit the central long axis of the scaphoid and 2 mm below the articular plane at both the proximal and distal ends, when setting cannulated screw into the scaphoid, it is easy to cause local reset loss and axial instability [21]. (4) Dexterously crossing 2 ASCs in fracture fixation prevents rotational displacement of the fracture fragments, which is, in terms of anti-torsion. This allows patients to undertake wrist function exercises at an early stage of healing and synchronously achieve fracture healing and functional recovery.

Beyond that, the limitations of these techniques are also obvious: (1) the invasive operation of iliac bone graft is inevitable in this procedure, and the pain after autologous iliac bone transplantation accounted for more than a third of all complications in the donor area, and the pain releases significantly in 3 years after surgery [22]. 12/18 of the patients felt pain in the follow-up of the 4th week, but only two patients aggravated the pain who needed oral NSAIDs to alleviate the symptoms for 4–6 weeks. We think transplantation of the iliac crest medial plate as well as less soft tissue dissection may help to relieve the pain in donor region; (2) if the fracture fragment is too small to find appropriate ASC to meet the requirements of solid fixation, it is easy to cause failure of fixation; (3) direct reduction via the volar approach has less damage to the blood supply, but it is inconvenient to observe dorsal angulation and displacement, and the degree of desorption of sclerosed bone is not easy to determine, which may lead to excessive desorption and difficult fixation.

However, there are several limitations in this study. This was a clinical experience summary with a relatively small number of cases and the lack of a control group. Further case–control studies with large sample numbers will increase the statistical power of this technology and promote the use of ASC in the treatment of scaphoid fracture nonunion.

## Data Availability

Not applicable.

## Abbreviations

Ni–Ti: nickel–titanium
ASC: arched shape-memory connector
